# Application of Unsupervised Transfer Technique Based on Deep Learning Model in Physical Training

**DOI:** 10.1155/2022/8679221

**Published:** 2022-04-14

**Authors:** Quanbin Zhao, Hanqi Wang

**Affiliations:** Department of Leisure Sports Teaching and Research Office, Shenyang Sport University, Shenyang 110102, Liaoning, China

## Abstract

The research purpose is to study the standardization and scientizing of physical training actions. Stacking denoising auto encoder (SDAE), a BiLSTM deep network model (SDAL-DNM) (a kind of training action model), and an unsupervised transfer model are used to deeply study the action problem of physical training. Initially, the physical training action discrimination model adopted here is a combination of stacked noise reduction self-encoder and bidirectional depth network model. Then, this model can collect data for five actions in physical training and further analyze the importance of action standardization for physical training. Afterward, the SDAL-DNM implemented here fully integrates the advantages of SDAE and BiLSTM. Finally, the unsupervised transfer model adopted here is based on SDAL-DNM deep learning (DL). The movement data of the physical training crowd are collected, and then the unsupervised transfer model is trained. According to the movement characteristics of physical training, the data difference between trainers is calculated so that the actions of each trainer can be continuously adapted according to the model, and finally, the benefits of effectively distinguishing the training actions can be achieved. The research shows that before and after unsupervised learning, the average decline of the model used is 1.69%, while the average decline of extreme learning machine (ELM) is 5.5%. The conclusion is that the unsupervised transfer model can improve the discrimination accuracy of physical training actions and provide theoretical support to effectively correct mistakes in physical training actions.

## 1. Introduction

With the continuous development of science and technology, technology in all aspects of society is constantly improved, and people's lifestyles have undergone earth-shaking changes. With the continuous improvement of material life, people began to take some physical exercises in their spare time. In this case, physical training [1–3] has become a new way of life. Sports can not only play the role of physical exercise but also promote the comprehensive development of people's spiritual quality. At present, there are various types of physical training in society. Nowadays, traditional physical training is no longer the only way of doing exercise in daily life [4–6] but also competitive physical training, recreational physical training, and medical physical training in the new era. Based on this, sports have become an indispensable part of life.

Relevant researchers have carried out a lot of research work according to the methods of physical training and the matching and adaptability of these methods to the human body. With the development of smart devices, portable and wearable smart devices are widely used. On this basis, the data collection of physical training [7–9] has provided great assistance to relevant researchers at home and abroad. Research shows that, based on the use of intelligent equipment, relevant scholars have confirmed that wearable devices based on embedded sensor networks can accurately detect the intensity of physical training and other indicators. Additionally, relevant scholars have studied the physical training with the random forest method of machine learning [10–12]. It is pointed out that this method can effectively distinguish various actions and behaviours in physical training on datasets. At present, the research on information retrieval of physical training action pictures is divided into two dimensions. On the one hand, it is the retrieval mode of physical training pictures based on text information. In this dimension, the image text database needs manual maintenance, each image needs manual description, and each image has more information, which brings more workload. On the other hand, it is the retrieval method of training action image information based on content, which is mostly used in the fields of shopping and industry. However, due to the large amount of processed data, there are high requirements for servers and developers. Jonker et al. [13] once pointed out that the main characteristics of image and text information processing for large-scale training actions are as follows: the data volume of action images is large, the feature dimension is high, and the response speed required by users is fast. However, the traditional action image retrieval algorithm is calculated based on similarity, which cannot adapt to the retrieval process of large-scale images at all. Nguyen et al. [14] once pointed out that the hashing algorithm has gradually become a research hotspot in image information retrieval because of its small storage and high retrieval efficiency. Research of Chang et al. [15] showed that the hash algorithm could greatly reduce the computation of training action image retrieval, but the algorithm also losed part of the information of action images, which reduced the performance of the model. As a research hotspot of machine learning, deep learning (DL) has obvious advantages in the field of physical training image detection due to its strong learning ability. Yu and He [16] found that DL was introduced into the field of image retrieval of physical training action. The feature detection efficiency of the image can be effectively improved. Although many scholars have carried out research in these two aspects, there are still some problems in the image retrieval of physical training motion; especially in the face of large-scale motion image information, the current algorithm is still difficult to calculate efficiently.

According to the current global research situation, as well as the existing problems and shortcomings, the unsupervised transfer method under the model of the DL algorithm is adopted to conduct in-depth research on various actions in physical training. Based on this, the combination is implemented on the effective discrimination of physical training actions and unsupervised transfer methods to study the research topic. The innovation is the combination of the unsupervised transfer method of the DL model with physical training for a brand-new study, which is of great practical significance for scholars engaged in research related to physical training.

## 2. Materials and Methods

### 2.1. Sports Action Discrimination Model

**Figure 1:**
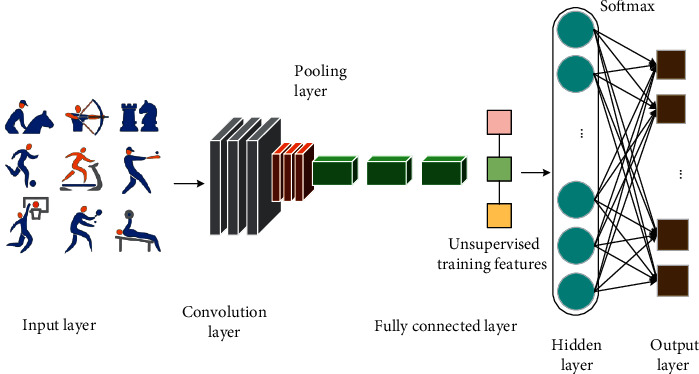
Frame of training action discrimination.

The model of physical training action discrimination here is a SDAL-DNM model. This model is used to collect data for five actions in physical training and further analyze the importance of action standardization for physical training. Wearable devices are used to collect data. SDAL-DNM constructed can fully integrate the advantages of SDAE and BiLSTM.  Figure 1 signifies the frame of training action discrimination.

One of the key points is that SDAL can connect previous information to the current task. In some cases, the current tasks can be performed only by the given previous information. SDAL can deal with such long-term dependency problems. People can carefully select parameters to solve the primary form of such problems. Yet, the traditional neural network model cannot successfully learn these knowledge points in practice. SDAL is deliberately designed to avoid long-term dependency problems. In practice, long short-term memory (LSTM) networks can remember the long-term information by default instead of obtaining information at great costs. By contrast, SDAL can remove or increase information to the cell state through a well-designed “gate” structure, a gateway for selective information passage. The gate contains a sigmoid neural network layer and a multiplication operation. For example, a language model might input a pronoun and output either another pronoun or verb-related information based on the input. Then, the model must consider the person and number of the input pronoun and know the inflectional verb rules to correct output information.

In the SDAL-DNM model, the loss function [17, 18] adopted is the cross-entropy loss. Its principle can be expressed as

(1) $$\begin{array}{c} \text{Loss}=-\sum_{i=1}^{n}y_{i}\log\left(y_{i}^{\prime }\right). \end{array}$$

Here, *y*_*i*_ and *y*′ refer to the true and the predicted values of training action data, respectively.

**Figure 2:**
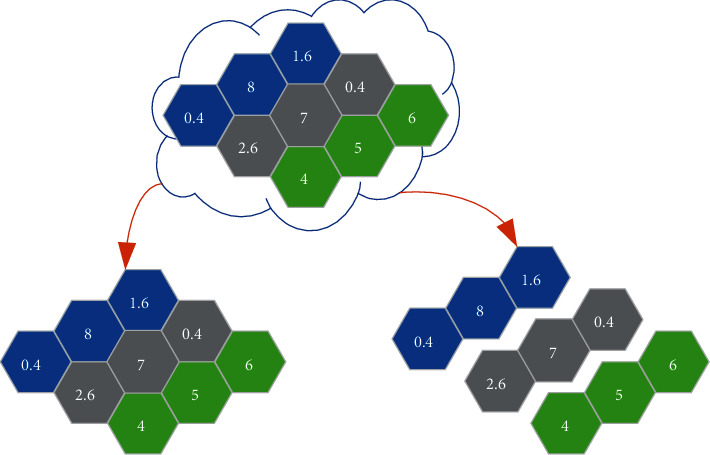
Data collected by sensors.

Deep convolutional neural network (DCNN) is a typical neural network structure, which is widely used in computer vision fields such as image recognition, target location, and face recognition. Local perception and weight sharing in convolution layer reduce the number of parameters and the complexity of the network model. Pooling reduces the size of feature graph and the number of parameters. There is always the problem of gradient disappearance when CNN activates the function with sigmoid, and especially in the complex network model, CNN is easy to overfit. Here, CNN is used to analyze and process the data collected during physical training.  Figure 2 shows the data collected by sensors.

Sensor detection is the frontier technology of modern science and technology. It is the basis of manufacturing automation and informatization. Meanwhile, it is a basic course suitable for college Majors, such as Electromechanical, Automation, Aviation, Navigation, and Aerospace. Sensor detection technology involves measuring, transforming, and processing various physical quantities, chemical quantities, and biomass. It is widely used and significant to industrial and agricultural production and national defense. For example, sports technological giants are developing smart clothing and smart insoles with the help of Internet of things (IoT) sensors to track the health and performance of athletes and collect sports data for analysis. An American sports equipment manufacturer has manufactured shoes with built-in IoT sensors to store and share data, such as athletes' running distance, speed, mileage, and fatigue. The data collected from sensors can be integrated with the team's internal systems to analyze athletes' performance, health status, stress, and injuries. Having realized the potential of the IoT in the sports fields, many enterprises invest heavily in advanced sports venues and sporting goods. However, the market of the IoT in the sports field is still quite primary. Therefore, sports institutions need to be prepared for IoT technological takeover. To do this, organizations can create their own strategies and build their infrastructure accordingly.

The deep feature extraction of SDAE is used to distinguish the training actions. Self-encoder consists of three parts: the first part is input layer, the second part is hidden layer, and the third part is output layer. Its training process is divided into two processes: coding and decoding. The process of feeding back the input data to the hidden layer is coding, and its process can be expressed as

(2) $$\begin{array}{c} y=f\left(W_{1}x_{i}+b_{1}\right). \end{array}$$

Here,  *x*_*i*_  is the output value. *W*_1_ and *b*_1_ represent weight and deviation. *g* stands for the sigmoid function.

(3) $$\begin{array}{c} z=g\left(W_{2}y+b_{2}\right). \end{array}$$

Here, *W*_2_ and *b*_2_ represent the weight and deviation. *g* is the sigmoid function. Here, the cross entropy loss can be applied to calculate the error between the initial data and the output data. Equation (4) expresses the detailed calculation.

(4) $$\begin{array}{c} L\left(x_{i},z\right)=-\sum_{i=0}^{n}\left(x_{i}\log\;z+\left(1-x_{i}\right)\log\left(1-z\right)\right). \end{array}$$

According to the results, updating can be realized on the overall network. Equation (5) is the conversion form when the coding layer performs coding operation.

(5) $$\begin{array}{c} y=f\left(W_{1}x_{i}^{\prime }+b_{1}\right). \end{array}$$

**Figure 3:**
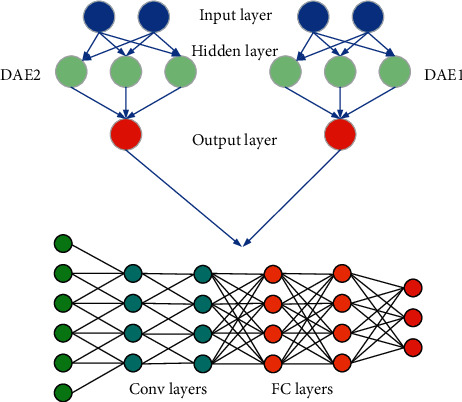
Schematic diagram of SDAE model structure.

A lot of noise reduction self-encoders are stacked to be turned into deeper networks. At this time, the SDAE network is obtained, and Figure 3 displays its structure.

### 2.2. Unsupervised Transfer Model

**Figure 4:**
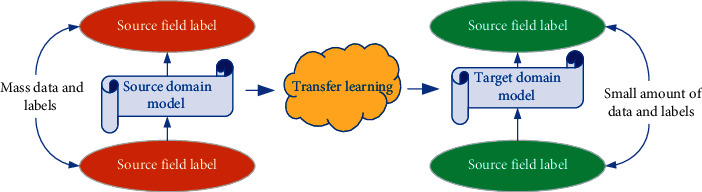
Working flow of transfer learning.

Transfer learning [19, 20] is actually a process of knowledge transfer in two different fields, and for new fields, transfer learning can be good assistance.  Figure 4 illustrates the specific flow of the learning.

**Figure 5:**
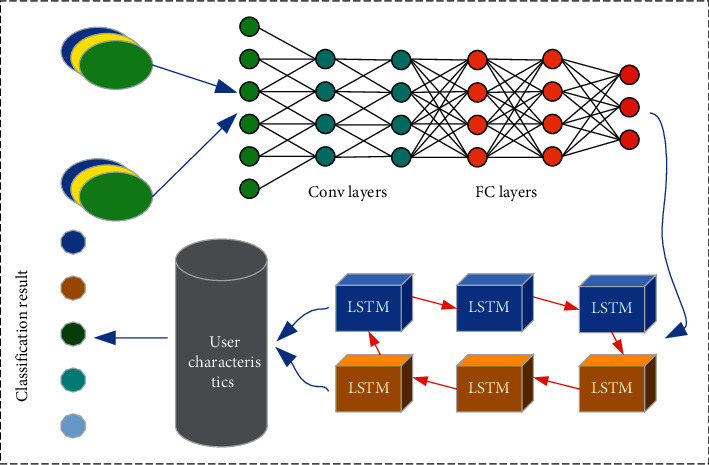
Working principle of unsupervised transfer model.

CNN can recognize the image scenes and provide relevant titles. It can also identify daily objects, humans, and animals. Recently, CNN has also played a great role in some natural language processing (NLP) tasks, such as sentence classification. Thus, CNN is becoming an important tool for most machine learning practitioners. Channel traditionally refers to a specific component of an image. Standard digital camera images have three channels (red (R), green (G), and blue (B)) and can be modeled as three stacked two-dimensional matrices mathematically, each with pixel values between 0 and 255. First, the image input mechanism into the neural network must be understood. Since the computer is more suitable for matrix operation than humans, scientists try to model images into matrices. All color images are superimposed by three RGB channels stored in the computer through three matrices. Then, the computer visualization technique presents layers with different shapes or structures. When these images are input to a CNN, these differently shaped and structured layers become different filters or operators. Then, each CNN neuron will select an operator to extract the input image features of the previous layer to get a stronger and higher-level representation. Therefore, compared with ordinary neural networks, CNN's every layer has a different mathematical representation. Yet, no matter how its mathematical representation changes, there is always a way to calculate its gradient using the piecewise function or convolution layer derivation functions. Only the convolution layer derivation functions might be more complex. The unsupervised transfer model here is based on SDAL-DNM DL. With this foundation, the movement data of the physical training crowd are collected to train the SDAL-DNM unsupervised transfer model. According to the movement characteristics of physical training, the data difference between trainers is calculated. Furthermore, the actions of each trainer can be constantly self-adapted according to the model, and finally, effectively distinguishing the training actions can be achieved.  Figure 5 explains the working principle of the unsupervised transfer model.

**Figure 6:**
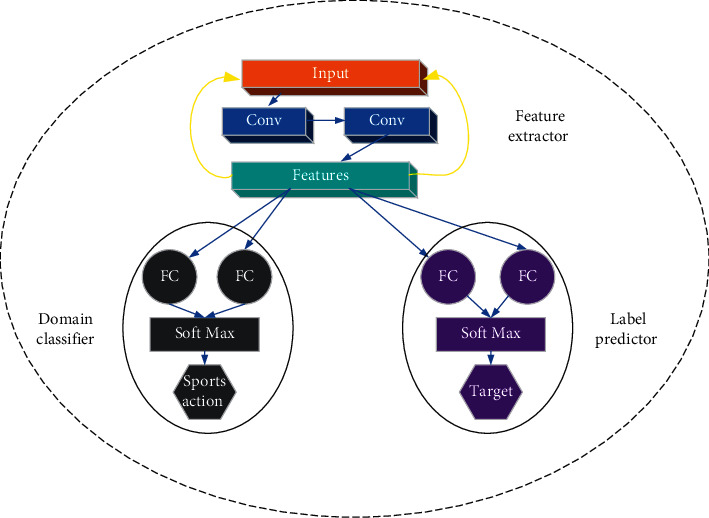
Schematic diagram of feature point extraction process.

In the unsupervised transfer learning of action discrimination for the process of physical training [21], invariant features are extracted according to the ambiguous features of target and source, of which data are further classified.  Figure 6 is a schematic diagram of the model structure for feature point extraction.

Generally speaking, the status of the system of unsupervised transfer is determined by *k*(1,2,3,…, *n*) time series, and the system model equation is as follows:

(6) $$\begin{array}{c} x_{k}=f_{k}\left(x_{k-1},v_{k}\right). \end{array}$$

Here, *x*_*k*_ represents the action state at time *k*. *f*_*k*_ is the state function at time *k*. *v*_*k*_ is the action state at time *k*. Generally, the action states meet the Gaussian distribution. In order to realize physical training or training action prediction, observation and analyzation of the state in the training process need to be realized in various ways. The measurement of its system can be expressed as

(7) $$\begin{array}{c} y_{k}=h_{k}\left(x_{k},n_{k}\right). \end{array}$$

Here, *y*_*k*_ represents the body state at time *k*. *h*_*k*_ is the state transfer function. *n*_*k*_ denotes the observed state, which normally tallies with Gaussian distribution and is independent from the training action *v*_*k*_. Based on Bayesian estimation, after the observation system data *y*_1:*k*_ is obtained, the prior knowledge *p*(*x*_*k*_*|x*_*k*−1_) is applied to estimate the state of the system, which includes prediction and update. The observed value of the system *y*_1:*k*_ is used to deduce the probability density of the current state of the system *p*(*x*_*k*_*|y*_1:*k*_).

(8) $$\begin{array}{c} p\left(x_{k}|y_{1:k}\right)=\frac{p\left(y_{1:k}|x_{k}\right)p\left(x_{k}|y_{1:k-1}\right)}{y_{1:k}}, \end{array}$$

(9) $$\begin{array}{c} p\left(x_{k}|y_{1:k-1}\right)=\int p\left(x_{k},x_{k-1}|y_{1:k-1}\right)\text{d}x_{k-1}, \end{array}$$

(10) $$\begin{array}{c} p\left(x_{k},x_{k-1}|y_{1:k-1}\right)=\int p\left(x_{k},x_{k-1}\right)p\left(x_{k-1}|y_{1:k-1}\right)\text{d}x_{k-1}, \end{array}$$

(11) $$\begin{array}{c} y_{1:k}=\int p\left(y_{k}|x_{k}\right)p\left(x_{k}|y_{1:k-1}\right)\text{d}x_{k}. \end{array}$$

**Figure 7:**
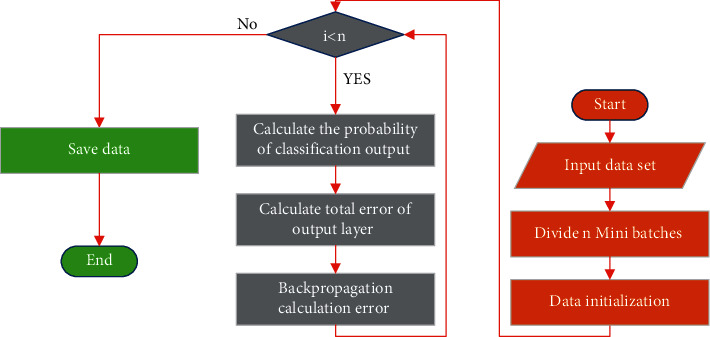
Flow of the algorithm.

Through equations (8)–(11), the recursion operation from time *k* to time *k* − 1 is obtained, but the concrete data value of probability analysis on the difficult movements in physical training is difficult to calculate, which leads to the impossibility of realizing the recursion operation. Therefore, the DL algorithm is introduced to predict physical training actions. The flow of the DL algorithm is as follows: Step 1: data are collected, and the convolution kernel and weights are randomly initialized. Step 2: according to the characteristics of forward propagation, relevant operations are carried out to calculate the output probabilities of multiple classification at the same time. Step 3: the output probability is compared with the actual physical training to calculate the error between them. Step 4: the gradient descent algorithm is used to reduce the loss in propagation.  Figure 7 signifies the detailed flow of the algorithm.

The CNN algorithm in DL [22–24] is used to extract features from the data collected in physical training. CNN can minimize the occurrence of preprocessing and can directly extract the most expressive features from the original data input by specifying features manually. In CNN, the main function of the pooling layer is to implement downsampling operation on the input feature map in two dimensions: length and width. It can reduce the number of parameters in the physical training model by implementing the downsampling operation on the input characteristic data. According to the index of the number of parameters, the data complexity of different actions in physical training is reduced. It can reduce the degree of overfitting of the neural network and the probability of falling into local minimum. In addition, the pooling layer can enhance the robustness of the model to translation and distortion in images.

According to the knowledge of DL, in the model of CNN [25–27], the action data of physical training go through multiple convolution layers and pooling layers, and then they are connected with each other by one or more full connection layers. In order to improve the network performance, ReLU function is usually used in the excitation function used by all neurons. Meanwhile, in order to reduce the amount of computation and enhance CNN's generalization performance, AlexNet (a model of deep CNN [28–30] with more network layers and stronger learning ability) is chosen. Further improvement is made on the functional layer of the convolution layer of the AlexNet model, from “local normalization and then pooling” to “pooling and then local normalization.” This improvement has two main advantages: first, it can further enhance the generalization ability of the AlexNet [31–33] network, weaken the phenomenon of overfitting, and greatly reduce the training time. Secondly, overlapping pooling before local normalization can not only keep more data information and weaken redundant information in the pooling process but also accelerate the convergence rate of the training action judgment prediction model in the physical training process. It can highlight the advantages of overlapping maximum pooling over previous maximum pooling methods.

In the improved AlexNet [34–36] algorithm flow, *λ*_3_=*λ*_4_ and *s* are initialized as

(12) $$\begin{array}{c} \lambda_{3}=\lambda_{4}=\frac{1}{n}\sum_{i=2}^{n}\left(\frac{k}{2}d_{i,k+1}^{x}-\frac{1}{2}\sum_{j=1}^{k}d_{ij}^{x}\right),\\ s_{ij}=\left\{\begin{array}{cc} 0, & x^{j}\notin N_{k}\left(x^{i}\right),\\ \frac{d_{i,k+1}^{x}-d_{ij}^{x}}{kd_{i,k+1}^{x}-\sum_{j=1}^{k}d_{ij}^{x}}, & x^{j}\in N_{k}\left(x^{i}\right). \end{array}\right) \end{array}$$

Here, *d*_*ij*_^*x*^=‖*x*^*i*^ − *x*^*j*^‖_2_^2^, *d*_*ij*_^*x*^ is sorted in ascending, in order to find out the smallest top *kd*_*ij*_^*x*^*s* among them. And, *N*_*k*_(*x*^*i*^) refers to the nearest *k* samples from *x*^*i*^. *F*^(*t*)^ is updated according to the equation as follows:

(13) $$\begin{array}{c} \underset{F}{\text{min}}\text{tr}\left(F^{T}L_{s}F\right)\\ \text{s.t.}\;F\in R^{n\times c},F^{T}F=I. \end{array}$$

The eigenvectors corresponding to the smallest *c* eigenvalues of Laplace matrix *L*_*s*_ can be used to form function *F*. Through equation (13), *s*^(*t*)^ can be solved.

(14) $$\begin{array}{c} \underset{s_{i}1=1,s_{i}\geq 0}{\text{min}}\sum_{i=1}^{n}\left(\lambda_{2}{\left\|w^{T}x^{i}-w^{T}x^{j}\right\|}_{2}^{2}s_{ij}+2\lambda_{3}s_{ij}^{2}\right)+\sum_{i=1}^{n}\lambda_{4}\left\|f_{i}-f_{j}{\;}_{2}^{2}\right\|s_{ij}. \end{array}$$

The physical training features are extracted more deeply. The *t*_th_ feature map *y*_*t*_^*l*^(*i*, *j*) of the *l*_th_ convolution layer is sampled in the way of overlapping pooling.

(15) $$\begin{array}{c} a_{t}^{l}\left(i,j\right)=\max\left\{y_{t}^{l}\left(i,j\right),i_{s}\leq i\leq i_{s}+w_{c}-1,j_{s}\leq j\leq j_{s}+w_{c}-1\right\}. \end{array}$$

Here, *s* represents the moving step of the pooling layer, and *w*_*c*_ indicates the width of the pooling layer *w*_*c*_ > *s*.

After the first and second pooling layers of the AlexNet [37] model, local normalization layers are added to standardize the feature map *c*_*t*_^*l*^(*i*, *j*).

(16) $$\begin{array}{c} c_{t}^{l}\left(i,j\right)=\frac{a_{t}^{l}\left(i,j\right)}{{\left(k+\alpha \sum_{\max\left(0,t-m/2\right)}^{\min\left(N-1,t+\left(m/2\right)\right)}{\left(a_{t}^{l}\left(i,j\right)\right)}^{2}\right)}^{\beta }}. \end{array}$$

Here, *k*, *α*, *β*, *m* are all superparameters, which can be set as 2, 0.78, 10^−4^, and 7 in turn. *N* stands for the total number of convolution cores in the *l*_th_ convolution layer. In order to prevent the problem of “gradient dispersion” in the network model [38], the ReLU function is used as the activation function for convolution output *S*_*t*_^*l*^(*i*, *j*).

(17) $$\begin{array}{c} y_{t}^{l}\left(i,j\right)=f\left(S_{t}^{l}\left(i,j\right)\right)=\max\left\{0,S_{t}^{l}\left(i,j\right)\right\}. \end{array}$$

Among parameters in equations (16) and (17), *S*_*t*_^*l*^(*i*, *j*) refers to the ReLU activation function. If the weight is set as *θ*=[*K*_*t*_^*l*^, *W*_*i*_^*l*^, *b*_*t*_^*l*^, *b*_*i*_^*l*^], and the gradient is expressed as *g*=[∂*L*/∂*K*_*t*_^*l*^, ∂*L*/∂*W*_*i*_^*l*^, ∂*L*/∂*b*_*t*_^*l*^, ∂*L*/∂*b*_*i*_^*l*^], then the biased first-order estimation *s* and second-order estimation *r* of the updated gradient can be calculated as follows:

(18) $$\begin{array}{c} s=\rho_{1}s+\left(1-\rho_{1}\right)g, \end{array}$$

(19) $$\begin{array}{c} r=\rho_{2}s+\left(1-\rho_{2}\right)g. \end{array}$$

In equation (18) and (19),  *ρ*_1_, *ρ*_2_ means estimated instantaneous exponential decay rates, 0.9 and 0.999 by default. Deviations $\hat{s}$ and $\hat{r}$ of the corrected first moment *s* and second moment *r* can be separately expressed as follows:

(20) $$\begin{array}{c} \hat{s}=\frac{s}{1-\rho_{1}^{t}},\\ \hat{r}=\frac{r}{1-\rho_{2}^{t}}. \end{array}$$

Finally, the updated weight *θ* is obtained.

(21) $$\begin{array}{c} \Delta \theta =-\varepsilon \frac{\hat{s}}{\sqrt{\hat{r}}+\delta },\;\theta =\theta +\Delta \theta . \end{array}$$

**Figure 8:**
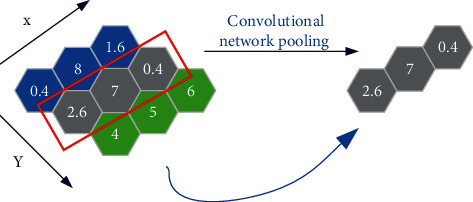
Schematic diagram of convolutional network pooling.

Here, *ε* represents a learning step. *δ* is a constant (10^−8^ by default).  Figure 8 is the schematic diagram of convolutional network pooling.

In order to prevent overfitting, the parameter of dropout operation is set to 0.5. When *l* equals 5 in equation (15), all feature images are reconstructed into a high-dimensional single-layer neuron structure *C*^5^; then, calculation can be made on the input *Z*_*i*_^6^ of the *i*_th_ neuron in the 6_th_ fully connected layer as

(22) $$\begin{array}{c} Z_{i}^{6}=W_{i}^{6}C^{5}+b_{i}^{6}. \end{array}$$

Here, *W*_*i*_^6^ and *b*_*i*_^6^ are the weight and offset of the *i*_th_ neuron in layer 6.

In the process of improving generalization ability, the neuron *C*^*l*^ is output without weight in the 6_th_ and 7_th_ fully connected layers. If $r_{j}^{l}∼\text{bernoulli}\left(dp\right),{\tilde{C}}^{l}=r^{l}C^{l}$, then the input *Z*_*i*_^*l*+1^ of the *i*_th_ neuron in the 7_th_ and 8_th_ fully connected layers can be represented by $W_{i}^{l+1}{\tilde{C}}^{l}+b_{i}^{l+1}$. And the output *C*_*i*_^*l*^ of the *i*_th_ neuron in the 6_th_ and 7_th_ fully connected layers can be referred by *f*(*Z*_*i*_^*l*^) or max{0, *Z*_*i*_^*l*^}. Finally, the input *q*^*i*^ of the *i*_th_ neuron in the 8_th_ fully connected layer can be obtained through the equation as follows:

(23) $$\begin{array}{c} q^{i}=\text{softmax}\left(Z_{i}^{8}\right)=\frac{e^{Z_{i}^{8}}}{\sum_{j=1}^{12}e^{Z_{i}^{8}}}. \end{array}$$

Meanwhile, the cross-entropy loss [39] suitable for classification task is used as the error function of the model, and its equation is as follows:

(24) $$\begin{array}{c} \text{Loss}=\sum_{i=1}^{K}y_{i}\cdot \;\log\left(p_{i}\right),\\ p_{i}=\frac{\exp\left({\tilde{y}}_{i}\right)}{\sum_{i=1}^{K}\exp\left({\tilde{y}}_{j}\right)}, \end{array}$$

where *k* is the number of categories. *y*_*i*_, ${\tilde{y}}_{i}$, and *p*_*i*_ represent the sample's actual category distribution, network output, and classification result. The input of the softmax function is an *N*-dimensional real number vector, which is set as *x*, and the equation is as follows:

(25) $$\begin{array}{c} \xi {\left(x\right)}_{i}=\frac{e^{x_{i}}}{\sum_{n=1}^{N}e^{x_{i}}},\;i=1,2,\ldots ,N. \end{array}$$

At the perspective of the essence, softmax function can map an *N*-dimensional arbitrary with real vector to an *N*-dimensional vector in which the values of each element are in the range of (0,1), thus realizing the normalization of the vector. In order to reduce the computational complexity of the model system, the compression-expansion conversion *μ* reduces the output data amount to 2^8^, i.e., *μ*  = 255, to improve the prediction efficiency of the model.

(26) $$\begin{array}{c} f\left(x_{t}\right)=\text{sign}\left(x_{t}\right)\frac{\ln\left(1+\mu \left|x_{t}\right|\right)}{\ln\left(1+\mu \right)},\;\left|x_{t}\right|<1. \end{array}$$

### 2.3. Dataset Collection

Firstly, the SDAL-DNM model collects five sporting processes of athletes: forward motion, backward motion, left motion, right motion, and squat motion. The model can capture and calculate athletes' acceleration in the sporting process. Then, through unsupervised transfer learning, the motion accuracy of athletes is captured and analyzed by extracting invariant features from the fuzzy features of targets and sources. The obtained results are sorted and statistically analyzed through visualization. Finally, the data are analyzed to demonstrate the research conclusions.

## 3. Results

### 3.1. Analysis of Training Action Model Results

**Figure 9:**
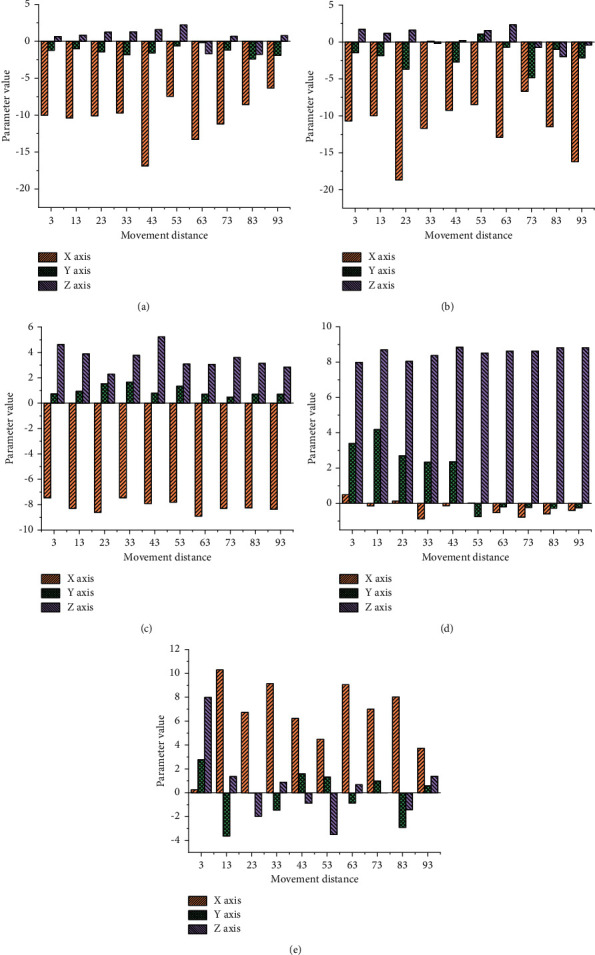
Acceleration curves of various actions: (a) forward movement; (b) move backward; (c) move to the left; (d) move to the right; (e) squat exercise.

The data of five training actions are collected and analyzed by the SDAL-DNM model.  Figure 9 presents the specific data of acceleration in three dimensions of different training actions. Among them, the acceleration change of *x* axis in Figure 9(a) is the largest, and the absolute value of the maximum value reaches 16.9 m/s^2^; Figure 9(b) also indicates that the acceleration of the *x* axis changes the most, with a specific value of 18.7 m/s^2^; the *x*-axis acceleration changes of Figures 9(c)–9(e) are not as good as those of Figures 9(a) and 9(b).  Figure 9 shows the acceleration curves of various actions.

### 3.2. Result Analysis of Unsupervised Transfer Model

**Figure 10:**
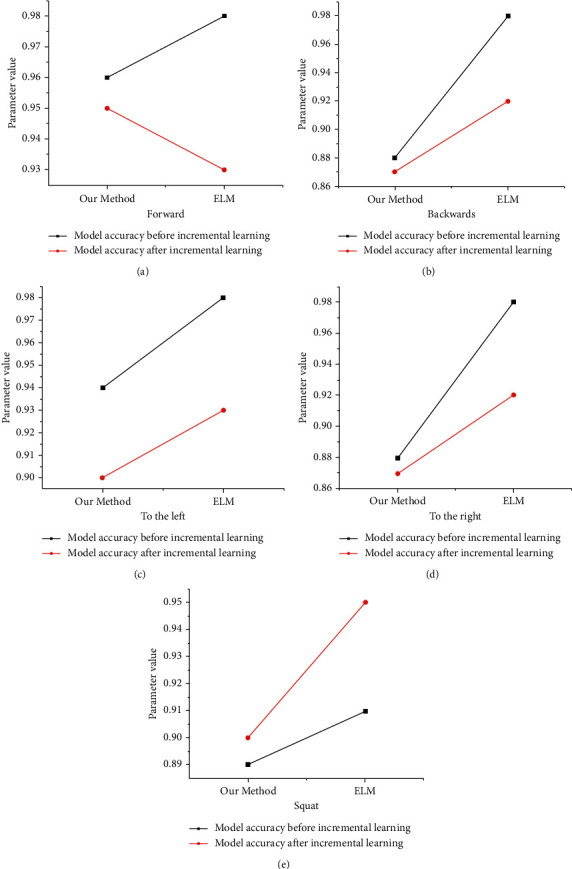
Comparison of model accuracy before and after learning: (a) moving forward; (b) move backward; (c) move to the left; (d) move to the right; (e) squat exercise.

The specific data collected here represent the concrete situation of every movement in the physical training. After the unsupervised transfer model, the specific analysis results are presented in Figure 10. In Figure 10(a), according to the accuracy before and after learning by using the research method, the average decline of the model studied is 1.69%, while that of extreme learning machine (ELM) is 5.5%. According to the situation of Figures 10(b)–10(e), the model has the lowest average decline. This fully demonstrates that the stability and transfer ability of the model adopted in this paper are superior to those of ELM. Figure 10 signifies the comparison of model accuracy before and after learning.

ELM is a feedforward neural network. It does not need gradient-based backpropagation to adjust the weight but sets it through Moore–Penrose generalized inverse. A series of calculations are completed by multiplying the input by the weight, adding the offset, calculating the activation function, calculating the output, and error backpropagation. The gradient descent takes a longer training time than the matrix inversion, and the accuracy and number of nodes are most critical of these calculated results. Then, in the first two datasets, different sizes of BP networks are used to obtain the same results as ELM. In the first case, the BP network's size is five times the original, and in the second case, the size of the BP network is two times the original. The experimental results indicate that the ELM method can accurately approximate datasets. The training process of ELM can be reduced to a nonlinear optimization problem. When the activation function of the hidden layer node is differentiable, the input weight of the network and the threshold of the hidden layer node can be randomly assigned. Given the defects of ELM, now more scholars are trying to improve the ELM using the variable scale optimization algorithm of compound chaos to obtain better generalization performance and higher prediction accuracy in one-step and multistep prediction problems. It might work to use ELM as the experimental technical support for the present work because it can accurately learn and calculate the athletes' movement speed under specific circumstances. However, ELM cannot capture the athletes' movements effectively, so the present work does not choose ELM.

## 4. Conclusions

Today, with the rapid development of science and technology, people's lifestyles are constantly changed. Meanwhile, physical training, as a way to exercise, has gradually become the trend of the new era. Under this background, relevant studies are carried out according to the physical training actions. Aimed at the accuracy of action discrimination in physical training, the SDAL-DNM model and the unsupervised transfer model are put forward, and the standardization and scientizing of physical training actions are deeply studied. By collecting the action data of physical training lovers, the unsupervised transfer model is implemented and the data differences among different trainers are calculated so that the actions of trainers can be adjusted adaptively, thereby achieving the effect of efficiently identifying training actions. According to the experimental results, the average decline of the model before and after unsupervised learning is about 3.8% less than that of ELM, which shows that the model proposed here has obvious advantages in performance. The final conclusions are as follows: (I) unsupervised transfer model can improve the discrimination accuracy of physical training actions and provide theoretical support for effective action-correction measures. (II) the SDAL model can effectively analyze the athletes' instantaneous state data and accurately capture and analyze their acceleration or real-time speed. The deficiency lies in the lack of training movement data collected, which has a certain influence on the universality and rigor. In future research, this is what needs to be paid attention to.

## Data Availability

The data used to support the findings of the study are included within the article.
